# Evaluation of *Potato virus X* mild mutants for cross protection against severe infection in China

**DOI:** 10.1186/s12985-019-1143-7

**Published:** 2019-03-20

**Authors:** Q. Q. Cong, Y. Wang, J. Liu, Y. F. Lan, Z. K. Guo, J. G. Yang, X.-D. Li, Y. P. Tian

**Affiliations:** 1 Shandong Province Key Laboratory of Agricultural Microbiology, Department of Plant Pathology, College of Plant Protection, Shandong Agricultural University, Tai’an, Shandong 271018 People’s Republic of China; 2 Key Laboratory of Tobacco Pest Monitoring Controlling & Integrated Management, Qingdao, 266101 China; 3 Tai’an Academy of Agricultural Sciences, Tai’an, 271000 Shandong China; 4 Heilongjiang Academy of Agricultural Sciences, Mudanjiang, 157011 Heilongjiang China

**Keywords:** Cross protection, *Potato virus X*, RNA dependent RNA polymerase, Virulence

## Abstract

**Background:**

Cross protection is a promising alternative to control plant viral diseases. One critical factor limiting the application of cross protection is the availability of attenuated mutants or mild strains. *Potato virus X* (PVX) infects many crops and induces huge economic losses to agricultural production. However, researches on the variability and mechanism of PVX virulence are scarce.

**Methods:**

The mutants were obtained by introducing mutations into the RNA dependent RNA polymerase (RdRp) gene of PVX via site-directed mutagenesis. Attenuated mutants were screen according to their symptoms in *Nicotiana benthamiana* plants. The protection efficacy against severe infection were evaluated with interval of 5, 10 and 15 days.

**Results:**

Among the 40 mutants obtained, four mutants carrying substitutions of either Glu^46^, Asn^863^, Asn^968^ or Glu^1001^ to Ala in PVX RdRp showed drastically attenuated symptom, accompanying with reduced accumulation levels of coat protein, plus- and minus-sense RNAs. When the interval between protective and challenging inoculations was 15 days, mutant E1001A (with substitution of Glu^1001^ to Ala in RdRp) provided complete protection against severe infection in both *Nicotiana benthamiana* and tomato, while E46A (Glu^46^ mutated to Ala) provided incomplete protection. To reduce the risk of reverse mutation, we constructed mutant dM which carries double mutations of both Glu^46^ and Glu^1001^ to Ala in RdRp. The mutant dM could provide effective protection against severe PVX infection.

**Conclusion:**

Mutations of Glu^46^, Asn^863^, Asn^968^ or Glu^1001^ to Ala in PVX RdRp significantly reduced the viral symptoms. Mutants E1001A and E46A could provide effective protection against wild type PVX in both *Nicotiana benthamiana* and tomato. These results provide theoretical and practical bases for the control of PVX via cross protection.

## Background

Cross protection is a phenomenon in which plants infected or pre-inoculated with one virus with mild symptoms will show resistance to the subsequent infection by a severe isolate of the same or closely related virus [1–3]. It was first described with *Tobacco mosaic virus* (TMV) by McKinney in 1929 [4]. Since then, cross protection has been used to control viruses including *Bean yellow mosaic virus* [5], *Citrus tristeza virus* [6, 7], *Clover yellow vein virus* [5], *Cucumber green mottle mosaic virus* [8], *Cucumber mosaic virus* [9–11], *East African cassava mosaic virus-Uganda* [12], *Papaya ringspot virus* [13, 14], *Pepipo mosaic virus* [15], *Pepper mild mottle virus* [16], *Soybean mosaic virus* [17], *Tobacco mosaic virus* [5, 18], *Tomato mosaic virus* [2, 19–22], *Watermelon mosaic virus* [9], and *Zucchini yellow mosaic virus* [9, 23, 24].

The pre-inoculated strains used for protective inoculation have been selected from naturally occurring isolates that cause mild symptoms [6, 9], by random or direct mutagenesis of wild severe strains using nitrous acid or ultraviolet irradiation [8, 21, 22], and by cultivation at higher or lower temperature than the optimal [5, 19, 23]. However, it is time consuming to screen the mild strains for cross protection. With the advent of reverse genetics, one can obtain mutants with site-directed mutagenesis, and then study their phenotypes and evaluate their potential in cross protection [16, 24]. This provides a faster, more effective and controllable way for screening attenuated strains.

*Potato virus X* (PVX; genus *Potexvirus*, family *Alphaflexiviridae*) can infect a wide range of major crops in the family *Solanaceae* including tomato, potato, pepper and tobacco. It can be transmitted by mechanical inoculation and contact between plants [25]. PVX has a positive-sense single-stranded (ss) RNA of 6435 nucleotides (nt), with a cap at the 5′-end and poly(A) tail at the 3′-end, respectively [26–29]. Its genome contains five open reading frames (ORFs). ORF1 encodes the replication associated protein RNA-dependent RNA polymerase (RdRp) [26, 30], while the overlapping ORFs 2, 3 and 4 encode the triple gene block (TGB) proteins (TGBp1, TGBp2 and TGBp3) which are essential for virus movement [31, 32]. ORF5 encodes the coat protein (CP) [33]. PVX isolates are classified into four groups based on their responses in the potato cultivars carrying *Nb*, *Nx* or *Rx* genes [34, 35], and two molecular groups, Eurasia and America, based on their complete genomic sequences [28]. Planting resistant cultivars is the most economic and effective way to control PVX. However, breeding virus resistant cultivars is time consuming and the resistance could be overcome by single amino acid mutation in CP or emerging of new strains [36, 37]. Therefore, cross protection become a promising alternative strategy for PVX control.

One prerequisite for cross protection is the availability of mild strains. Salaman (1933) reported the application of mild strains in cross protection against PVX [38]. However, no PVX mild strain is available in practice. Moreover, the mechanisms regulating the virulence of PVX are largely unknown. There are several reports in which viral symptom determinants have been mapped to the silencing suppressors [24, 39–43] or the replicases [44–47]. Mutation in these proteins may abolish the RNA silencing suppressor activity and reduce viral symptoms. Some mutants carrying such mutations were reported to confer cross protection against parental viral strain [24, 48]. The 25 kDa TGBp1 (P25) is an RNA silencing suppressor and plays an important role in the movement of PVX [31, 32, 49]. We firstly tried to screen attenuated PVX mutants by introducing mutation to TGB region encoding P25. However, all the mutants in TGBp1 either had movement defective or displayed severe symptom as severe as wild type PVX. Therefore, we failed to obtain any mild strain from TGBp1 mutants. In this paper, we obtained four attenuated PVX mutants by introducing single amino acid mutation to RdRp via site-directed mutagenesis, evaluated their potential in cross protection and elucidated the underlying mechanism; we also obtained one mutant with double mutations at two amino acid sites to increase its safety.

## Methods

### Mutant construction

The amino acid residues (aa) predicted by software I-TASSER [50] to be exposed on the surface of PVX RdRp were substituted with aa of opposite polarity. The asparagine residue in postulated glycosylation site was mutated to alanine. Mutations were introduced to wild type PVX infectious clone pCaPVX100 by site-directed mutagenesis using Phusion high-fidelity DNA polymerase (Thermo, Finland) and primers designed following the strategy reported previously (Table 1) [51, 52]. The fidelity of all mutants was verified by sequencing.

**Table 1:** Primers used for site-directed mutagenesis of PVX mutants

Mutant	Primer	Sequences
V45R	Forward	5′-CTCAAACGCGTGAAGCGGCTAATGATCTAGAGGGGTTCGGCATAG-3′
	Reverse	5′-GCCGCTTCACGCGTTTGAGCGTACGGGTTAGATAGTTTGTGTTTTTCC-3′
E46A	Forward	5′-TCAAACGGTTGCCGCGGCTAATGATCTAGAGGGGTTCGGCATAGC-3′
	Reverse	5′-ATTAGCCGCGGCAACCGTTTGAGCGTACGGGTTAGATAGTTTGTGTTTTTC-3′
T140A	Forward	5′-ACAGAGATCGCCACGGACACAGCATACATTAGTGACACTCTGCACTTC-3′
	Reverse	5′-GTGTCCGTGGCGATCTCTGTGAGCTTGTCTATTATTGTTTCCTTTGGG-3′
D200A	Forward	5′-TACTTTGGAGCCGGTTTCCAGTATATACCAGGCAACCATGGTGG-3′
	Reverse	5′-TACTGGAAACCGGCTCCAAAGTATTTGAGGCTGTATATGTTCGGGT-3′
N243A	Forward	5′-CTCGGACATCTCGCCTACACGACTGAGCAGGTTGAGATGCACACAG-3′
	Reverse	5′-AGTCGTGTAGGCGAGATGTCCGAGAAAGCTATCCTTGGGGTCCCTC-3′
K299A	Forward	5′-ATCTTTCTCCCGGCAGTTCACAACTGCAAGAAGCCGATTCTGAAGA-3′
	Reverse	5′-GTTGTGAACTGCCGGGAGAAAGATCTGTGGTGGAATCACATACCTGTCA-3′
V300R	Forward	5′-CTCCCGAAACGCCACAACTGCAAGAAGCCGATTCTGAAGAAAAC-3′
	Reverse	5′-CAGTTGTGGCGTTTCGGGAGAAAGATCTGTGGTGGAATCACATACC-3′
S341R	Forward	5′-ATTAAATCGCGCGACTTGGACAAATACTCTGCTGTGGAACTGGTTTAC-3′
	Reverse	5′-GTCCAAGTCGCGCGATTTAATTAATTGTCTGACTTTGGCAAAAATG-3′
D344A	Forward	5′-GTCTGACTTGGCGAAATACTCTGCTGTGGAACTGGTTTACTTAGTGAGC-3′
	Reverse	5′-GCAGAGTATTTCGCCAAGTCAGACGATTTAATTAATTGTCTGACTTTGGCA-3′
E446A	Forward	5′-TCGGACGTAGCCGAAATGGAAAGTTTGTTCTCAGATGGGGACCTG-3′
	Reverse	5′-TTTCCATTTCGGCTACGTCCGACACTTCCCTTGGTCGGAAGG-3′
A494K	Forward	5′-ATTAAAGAACCTAAAGGAGACAGAAATCAATACTCAAACCCTGCGGAA-3′
	Reverse	5′-TTCTGTCTCCTTTAGGTTCTTTAATTCCTTGACCGACATCCATCTCA-3′
K525A	Forward	5′-CAGACCACAGCGAAGGCTAAGCGCCTAGCTGAAATCCAGGAGTCC-3′
	Reverse	5′-GCTTAGCCTTCGCTGTGGTCTGATGTTTCACCTCTCTACTGTGTTTCCTGT-3′
K526A	Forward	5′-CAGACCACAAAGGCGGCTAAGCGCCTAGCTGAAATCCAGGAGTCC-3′
	Reverse	5′-GCTTAGCCGCCTTTGTGGTCTGATGTTTCACCTCTCTACTGTGTTTCCTGT-3′
K528A	Forward	5′-AAAGAAGGCTGCGCGCCTAGCTGAAATCCAGGAGTCCATGA-3′
	Reverse	5′-TAGGCGCGCAGCCTTCTTTGTGGTCTGATGTTTCACCTCTCTACTG-3′
E540A	Forward	5′-ATGAGAGCAGCCGGTGAAGCTGAATCAAATGAGATGAGCGGG-3′
	Reverse	5′-AGCTTCACCGGCTGCTCTCATGGACTCCTGGATTTCAGCTAGGC-3′
S545A	Forward	5′-GAAGCAAATGAGATGAGCGGGGGCATGGGGGCAATACCG-3′
	Reverse	5′-CCCGCTCATCTCATTTGCTTCAGCTTCACCTTCTGCTCTCATGGACT-3′
S565A	Forward	5′-AGCACGGCTGATGCTAGACAAGAACTCACACTCCCAACCACC-3′
	Reverse	5′-GTCTAGCATCAGCCGTGCTGGGAAGTTCAGCGTTGCTCG-3′
D566A	Forward	5′-AGCACGAGTGCCGCTAGACAAGAACTCACACTCCCAACCACCAA-3′
	Reverse	5′-TCTTGTCTAGCGGCACTCGTGCTGGGAAGTTCAGCGTTGCTCG-3′
M610K	Forward	5′-GAGACAGCAAAACAACAAGTCATCGAAGGACTCCCTTGGAAA-3′
	Reverse	5′-ACTTGTTGTTTTGCTGTCTCAACAGCTTCTTTTCCAGGGAGCTT-3′
K620A	Forward	5′-ACTCCCTTGGGCACACTGGATTCCTCAACTAAACGCTGTTGGATTC-3′
	Reverse	5′-AATCCAGTGTGCCCAAGGGAGTCCTTCGATGACTTGTTGCATTG-3′
N641A	Forward	5′-GGGATAGGGCTGGAACAATGATCATGCCTATCACAGAAATGG-3′
	Reverse	5′-ATTGTTCCAGCCCTATCCCTCTGAATTTCTAGCGCCTTGAATC-3′
E662A	Forward	5′-ACTTCCCGGCCGGAACTCCAAAAGAGTTGGCACGAGAATTGCTC-3′
	Reverse	5′-TGGAGTTCCGGCCGGGAAGTCCTCTTTTTCCAACCCGGAGA-3′
N676A	Forward	5′-TGCTCGCTATGGCGAGAAGTCCTGCCACCATCCCTTTGGAC-3′
	Reverse	5′-GGACTTCTCGCCATAGCGAGCAATTCTCGTGCCAACTCTTTTGGA-3′
K773A	Forward	5′-AGATTGGAGTGCGAAAGTGCCCAACACTGAACCATACATGTTCAAGA-3′
	Reverse	5′-GGCACTTTCGCACTCCAATCTAGCCGCAGTTCATTGGTCG-3′
N777A	Forward	5′-AAAGTGCCCGCGACTGAACCATACATGTTCAAGACCTATGAAAAGGCAT-3′
	Reverse	5′-GGTTCAGTCGCGGGCACTTTCTTACTCCAATCTAGCCGCAGTTC-3′
E779A	Forward	5′-CCAACACTGCCCCATACATGTTCAAGACCTATGAAAAGGCATTAATTGG-3′
	Reverse	5′-AACATGTATGGGGCAGTGTTGGGCACTTTCTTACTCCAATCTAGCCG-3′
N863A	Forward	5′-CGATACTATCTCGCCGCCACACACCGCAACAAGAAAGACCTTGC-3′
	Reverse	5′-TGTGTGGCGGCGAGATAGTATCGGCAGTATTTTGAGAAGTACTCTGTCGC-3′
N917A	Forward	5′-ACCGGAAGGGCCGACACGTTCACATACGCTGGATGCCAAG-3′
	Reverse	5′-ACGTGTCGGCCCTTCCGGTGCCCATGTACAGCTTTCTCTTTTCA-3′
N964A	Forward	5′-CACTTCGTGGCGACAAGTGCAAACTCTTCGGCCTTCTGGGAA-3′
	Reverse	5′-TGCACTTGTCGCCACGAAGTGAATCCTGTCGGTAGCTCTAGAGAGTGC-3′
N968A	Forward	5′-CACAAGTGCAGCGTCTTCGGCCTTCTGGGAAAAGTTAGACA GCACCC-3′
	Reverse	5′-GCCGAAGACGCTGCACTTGTGTTCACGAAGTGAATCCTGTCGGT-3′
E1001A	Forward	5′-AGCCGGCAGCCGTAGAGCCAATTCGAGAGCCTGAGCCC-3′
	Reverse	5′-GCTCTACGGCTGCCGGCTCGTACTCCTTGAGTGCTTGTTCTCTC-3′
R1006A	Forward	5′-AGCCAATTGCCGAGCCTGAGCCCCAAACACACATGTGTGT-3′
	Reverse	5′-TCAGGCTCGGCAATTGGCTCTACCTCTGCCGGCTCGTACTC-3′
E1041A	Forward	5′-ATCCACTCTGCCTCCCATGGCCATTCAAACTGTGTCCAAACTGA-3′
	Reverse	5′-CCATGGGAGGCAGAGTGGATCTCTCTGTCAAACTTTTCCAAAAGTTCC-3′
S1042A	Forward	5′-ACTCTGAAGCGCATGGCCATTCAAACTGTGTCCAAACTGAAGACACA-3′
	Reverse	5′-AATGGCCATGCGCTTCAGAGTGGATCTCTCTGTCAAACTTTTCCAAAAG-3′
K1065A	Forward	5′-TCAACAAGCAGCGGATGAGACCCTCCTCTGGGCGACCATAGATG-3′
	Reverse	5′-GGTCTCATCCGCTGCTTGTTGATGCGAAAA CAGCTGGATGGTT-3′
S1092A	Forward	5′-AATTCTTGGCCAAGAAGGACATTGGAGACGTCCTGTTTCTAAAC-3′
	Reverse	5′-GTCCTTCTTGGCCAAGAATTCTCGGAAGTTTGTTTCTTGATTG-3′
N1143A	Forward	5′-AACTTGATTGCGGGGACTGTGAGACAGAGCCCAGACTTTGAT-3′
	Reverse	5′-CACAGTCCCCGCAATCAAGTTGCACTTTGACTTACTGAGGTATTTG-3′
D1348A	Forward	5′-ACAGGCTTGAAGCGAAATTACTCCTCAAGTCGAAGCCTGTAATCACG-3′
	Reverse	5′-GGAGTAATTTCGCTTCAAGCCTGTGGAAACTAAGCTTCACTTCTGGAACG-3′
K1427A	Forward	5′-TTGTTCGATGAGGCGCAGTGTCAGGCACATACACTCACTTGCAG-3′
	Reverse	5′-ACACTGCGCCTCATCGAACAAGTCATGCAGAGAGTCCTTGTGGT-3′
L1456K	Forward	5′-CAGAAACTTTAAGTAACCGTTAAGTTACCTTAGAGATTTGAATAAGATGG-3′
	Reverse	5′-TAACGGTTACTTAAAGTTTCTGAGGCGGGGAAGTGAGACAGTGCCT-3′

Mutated nucleotides are shown in bold type

### Plant growth and virus inoculation

Plants of *N*. *benthamiana*, *N. tabacum* and tomato (*Solamum lycopersicum*) were maintained in a growth chamber with a 16 h light/8 h dark cycle and relative humidity of 70% at 23 ± 2 °C.

The infectious clone pCaPVX100 and its mutants were inoculated via the method previously described [52]. The *Agrobacterium tumefaciens* GV3101 cells were transformed following the freeze and thaw transformation procedure and cultured on solid LB medium at 28 °C. Agroinfiltration was performed with agrobacterium culture that was diluted in induction solution (10 mmol/L MES, pH 5.8, 0.15 mmol/L acetosyringone and 10 mmol/L MgCl_2_) to final optimal density of OD_600_ = 0.5. The first and second fully expanded leaves of 3- to 5-leaf stage *N*. *benthamiana*, *N. tabacum* and tomato plants were agro-infiltrated using a 1-mL needle-less syringe. From the sixth day post agro-infiltration (dpai), symptoms on the systemic leaves were recorded daily. Each mutant was inoculated to six plants and the experiments were repeated three times independently.

### Western blotting analysis

The accumulation of wild type and mutant PVX in *N. benthamiana* and tomato plants were determined using Western blotting. Total proteins were extracted from the systemic leaves of *N. benthamiana* and tomato plants, separated by 15% sodium dodecyl sulfate-polyacrylamide gel electrophoresis (SDS-PAGE), then transferred to a nitrocellulose (NC) membrane. The NC membrane was blocked with 5% defatted milk powder in pH 7.6 Tris-buffered saline containing 0.05% Tween-20 (TBST) for 1 h, incubated in antiserum against PVX CP diluted at 1:1000 (*V*/V) for 1 h, followed by 1 h incubation with alkaline phosphatase conjugated goat anti-rabbit IgG diluted in 1:50000 (Sigma) and finally colorized with NBT and BCIP.

### Qualitative detection of PVX

The presence of wild type and mutant PVX in plants was determined by reverse transcription-polymerase chain reaction (RT-PCR). Total RNAs were extracted from systemic leaves at 10 dpai using Transzol reagent (Transgen) according to the manufacturer’s instruction. The first-strand cDNA was synthesized using oligo (dT) primer and Moloney *Murine Leukemia Virus* (M-MLV) reverse transcriptase (Transgen). The cDNA products were used to amplify the PVX *cp* gene with specific primers PVXCP-F (5′-ATT GAG GCT ATC TGG AAG GA-3′) and PVXCP-R (5′-GCC TCA GCG GTT GTT GTT CC-3′). The PCR program included an initial denaturation at 94 °C for 5 min, 35 cycles of 30 s at 94 °C, 30 s at 55 °C, and 1 min at 72 °C, followed by a final extension of 10 min at 72 °C. The resultant PCR products were separated by electrophoresis on 1.0% agarose gel.

### Cross protection assay

To examine whether the attenuated mutants could provide protection against the wild type PVX, plants of *N. benthamiana* were agro-infiltrated with suspension of *Agrobacterium* cells containing the attenuated mutants. The plants infiltrated with *Agrobacterium* cells carrying empty vector pCAMBIA0390 were used as control. At 5, 10 and 15 days after the protective inoculation, the first fully expanded systemic leaves of the protected *N. benthamiana* plants were challenged with saps from wild type PVX-infected *N. benthamiana* plants by mechanical inoculation. The cross protection efficacy were evaluated by symptom development on the plants and the virus accumulations were determined by Western blotting assay using antisera against PVX CP. The same cross protection assays were conducted on *N. tabacum* and tomato plants.

### Genetic stability assay

The mutants including E1001A and dM were tested through successive passages to investigate the stability of both the mutations and the mild symptoms. *N. benthamiana* plants were first infiltrated with *Agrobacterium* cells carrying the attenuated mutants, and then the sap of leaves of systemic infection were collected to inoculate healthy *N. benthamiana* plants. The mutants were transferred every 10 days, successively transferred for 5 generations. Primers specific for PVX RdRp were used to clone the RdRp gene from the fifth generation of inoculated *N. benthamiana* plants.

### Northern blotting analysis

The accumulation levels of plus- and minus- strand RNAs in *N. benthamiana* plants inoculated with wild type and mutant PVX were analyzed by Northern blotting hybridization at 10 dpai. Total RNAs were extracted from systemic leaves using Transzol reagent (Transgen). Five μg of total RNAs was separated by 1.5% agarose formaldehyde gels and transferred to positively charged nylon membranes. The membrane was hybridized overnight at 65 °C with digoxigenin (DIG)-labelled RNA probes (DIG RNA Labelling Kit, Roche Molecular Biochemicals), followed by incubation with anti-DIG Fab fragments. The signal was detected using CDP-star as described in the DIG application manual. The minus- and plus-strand RNA specific probes synthesized separately by PCR amplification of PVX CP followed by in vitro transcription with T7 RNA polymerase. The upstream primer for minus-sense probe was 5′-*TAA TAC GAC TCA CTA TAG GG*A TGT CGT CAT CAG CTA GCA C-3′ which included nt 5649–5668 of the PVX genome and a T7 promoter (in italics). The downstream primer was 5′-ATG TAG ACG TAG TTA TGG TG-3′ which was complementary to nt 6374–6355 of the PVX genome. The upstream primer for the plus-sense probe was 5′-ATG TCG TCA TCA GCT AGC AC-3′ (identical to 5649–5668 nt in PVX genome), and the downstream primer 5′-*TAA TAC GAC TCA CTA TAG GG*A TGT AGA CGT AGT TAT GGT G-3′ was complementary to 6374–6355 nt in PVX genome, with the sequence in T7 promoter in italics.

To distinguish the small interfering RNAs (siRNAs) and RNAs of mild mutants from that of the wild type PVX, the saps of plants inoculated with *gfp-*carrying plasmid pCaPVX440GFP were used for challenging inoculation. Hence, the accumulation levels of siRNA and RNA from the wild type PVX can be determined using probes specific for the *gfp* gene. Ten days after pre-inoculation with attenuated mutants, the *N. benthamiana* plants were mechanically inoculated with saps from pCaPVX440GFP infected plants. Ten days later, total RNAs were extracted from the systemic leaves of challenged plants using Transzol reagent (Transgen) and analyzed for viral RNA accumulation by Northern blotting hybridization. The probes used to detect viral RNA were PCR-amplified *gfp* gene followed by in vitro transcription (Roche). The upstream primer for PCR amplification was 5′-GCG GTA CCA TGA GTA AAG GAG AAG AAC-3′. The downstream primer was 5′-*TAA TAC GAC TCA CTA TAG GG*T TTG TAG AGC TCA TCC ATG C-3′ which included a T7 promoter (in italics). The same plants were analyzed for the siRNA accumulation in response to virus inoculation using RNAiso for Small RNA kit (Takara). Ten μg of small RNAs were separated on a 15% TBE urea acrylamide gel and subjected to Northern blotting hybridization with probe described above. The membrane was hybridized overnight at 37 °C. The experiment was repeated three times independently.

## Results

### Effects of mutations in RdRp on virulence, CP and RNA accumulations of PVX

**Fig. 1:**
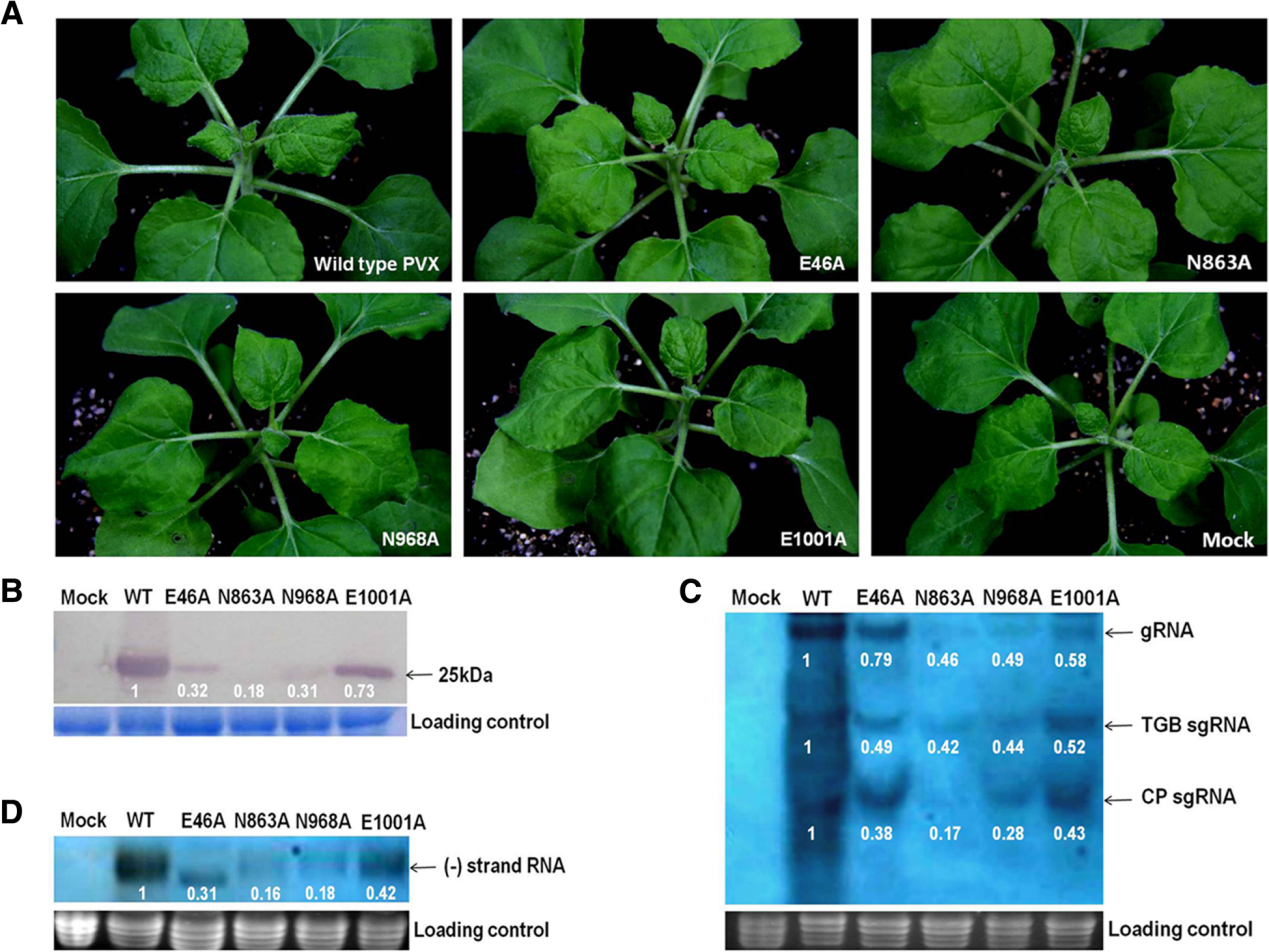
Symptoms and accumulation levels of wild type (WT) PVX and four attenuated mutants in *N. benthamiana* plants. **a** Symptoms of WT PVX and mutants E46A, N863A, N968A and E1001A at 8 days post agro-inoculation (dpai). Mock: Plants inoculated with *Argobacterium* cells carrying the empty vector pCAMBIA0390. **b** The CP accumulation level in the systemically infected *N. benthamiana* leaves at 8 dpai was determined by Western blotting. The relative CP accumulation level of each mutant was marked. **c and d** The accumulation levels of plus- (**c**) and minus-strand (**d**) RNA in *N. benthamiana* plants infected with WT PVX and four mutants at 10 dpai. The numbers in the figure represented the accumulation levels of each mutant relative to that of WT PVX. (+) strand RNA: PVX genomic RNA; TGB sgRNA: subgenomic RNA for triple gene block; CP sgRNA: subgenomic RNA for coat protein gene; (−) strand RNA: RNA complementary to PVX genomic RNA

In total, we obtained 40 PVX mutants with amino acid substitution in RdRp (Table 1). Most of these 40 mutants induced severe mosaic symptoms in *N. benthamiana* plants similar to that the wild type PVX, however, mutants E46A (Glu^46^ of RdRp mutated to Ala), N863A (Asn^863^ to Ala), N968A (Asn^968^ to Ala) and E1001A (Glu^1001^ to Ala) only induced inconspicuous symptoms on the leaves of inoculated *N. benthamiana* plants (Fig. 1a), indicating that mutations of Glu^46^, Asn^863^, Asn^968^ and Glu^1001^ in RdRp significantly reduce the virulence of PVX. The RT-PCR assay revealed that a 506 bp band specific to PVX CP was detected from the upper non-inoculated leaves of plants inoculated with any of these four mutants, showing that these four mutants could infect *N*. *benthamiana* plants systemically (data not shown).

In Western blotting assay, the CP accumulation levels of these four mutants in the systemic leaves of *N*. *benthamiana* plants were significantly lower than that of wild type PVX (Fig. 1b). The plus- and minus-strand RNA accumulation levels of PVX and the attenuated mutants in *N. benthamiana* plants were measured by Northern blotting hybridization. The results showed that the levels of plus-strand genomic RNA, TGB and CP sgRNAs in these mutants were lower than that of wild type PVX, and N863A had the lowest RNA accumulation level (Fig. 1c). The minus-strand genomic RNA accumulation levels of these four mutants in *N. benthamiana* plants were also lower than that of wild type PVX (Fig. 1d).

Taken together, these results indicated that mutations of Glu^46^, Asn^863^, Asn^968^ and Glu^1001^ in RdRp to Ala had significant impact on the virulence, the CP and RNA accumulation levels of PVX in the systemic leaves of *N. benthamiana* plants.

### Capacity of the attenuated mutants to confer protection against severe PVX infection

When the interval between protective and challenging inoculations was five days, plants pre-inoculated with the attenuated mutants E46A, N863A, N968A, E1001A or the empty vector pCAMBIA0390 showed severe mosaic symptom at 15 days after the challenge inoculation, indicating that an interval of five days is not enough for the attenuated mutants to elicit cross protection (data not shown).

**Fig. 2:**
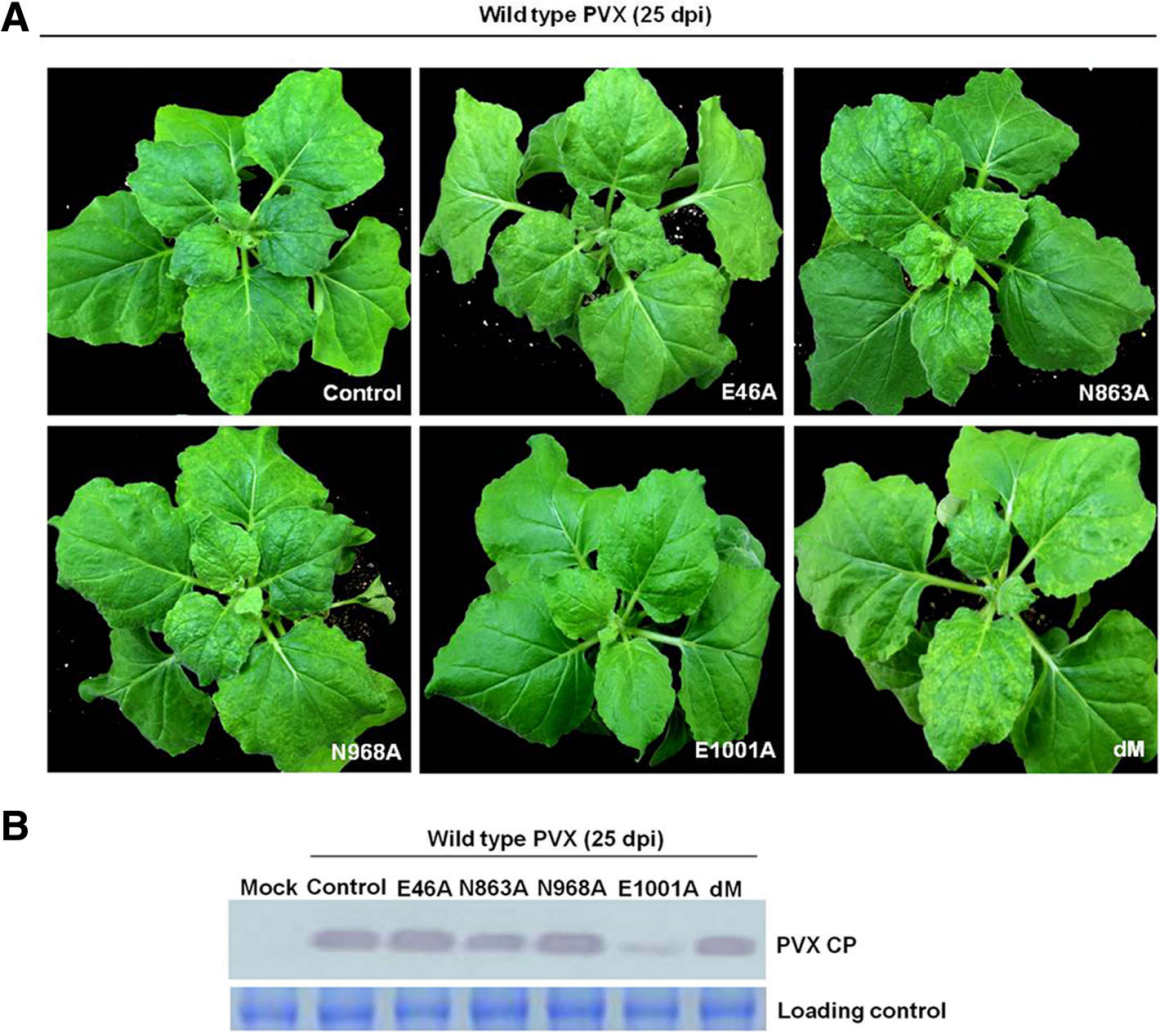
Cross protection of *N. benthamiana* plants against the severe infection with wild type PVX. **a** The protection effects of mutants E46A, N863A, N968A, E1001A and dM against wild type PVX at an interval of 10 days. The symptoms were photographed at 25 days after the challenging inoculation. Plants pre-inoculated with empty plasmid pCAMBIA0390 were used as control. The experiments were repeated three times independently (same below). **b** The accumulation of wild type PVX CP in E46A-, N863A-, N968A-, E1001A- and dM-protected systemically infected *N. benthamiana* leaves. The samples were detected by Western blotting using antiserum against PVX CP at 25 days after the challenging inoculation. Total RNAs were used as loading control

When the protective interval was increased to ten days, all the plants pre-inoculated with E1001A displayed no symptom and a low concentration of PVX CP was detected at 25 days after the challenging inoculation (Fig. 2a and b), indicating that E1001A could provide complete protection against severe PVX infection with an interval of ten days. Plants protected with E46A were absent of viral symptoms at 15 days post challenging inoculation, however, they showed severe mosaic symptoms and high concentration of PVX CP at 25 days post challenging inoculation (Fig. 2a and b), indicating that E46A could delay the infection of wild type PVX. All the plants pre-inoculated with N863A and N968A displayed obvious mosaic symptoms at 15 days after the challenge inoculation and high concentration of PVX CP were detected by Western blotting at 25 days after challenging inoculation (Fig. 2a and b), indicating that an interval of ten days is also not sufficient to elicit cross protection.

When the interval was increased to 15 days, all the *N. benthamiana* plants pre-inoculated with N863A and N968A showed distinct mosaic symptoms at 15 days post challenge inoculation. In total 12 of 18 *N. benthamiana* plants pre-inoculated with E46A showed viral symptoms. In contrast, all *N. benthamiana* plants pre-inoculated with E1001A showed no symptoms at 25 days post challenge inoculation (Table 2). These results indicate that mutant E46A could provide incomplete protection to PVX infection, while E1001A provide complete protection to PVX infection with a protective interval of 15 days. Similar results were obtained on the *N. tabacum* plants (Table 2).

**Table 2:** The protection efficacy of attenuated mutants against severe infection of PVX in *N. benthamiana* and *N. tabacum* plants

Mutants	*N. benthamiana*			*N. tabacum*
	5 days ^a^	10 days	15 days	15 days
E46A	18/18^b^	18/18	12/18	13/18
N863A	18/18	18/18	18/18	18/18
N968A	18/18	18/18	18/18	18/18
E1001A	18/18	0/18	0/18	0/18
dM	18/18	18/18	13/18	14/18
Control ^c^	18/18	18/18	18/18	18/18

^a^Protective intervals of 5, 10 and 15 days were tested for *N. benthamiana* plants, but only interval of 15 days was tested for *N. tabacum* plants

^b^Number of infected/protected plants. The infection was confirmed with Western blotting assay

^c^Control: The plants were pre-inoculated with empty vector pCAMBIA0390

### Protection efficacy of E1001A in tomato plants

**Fig. 3:**
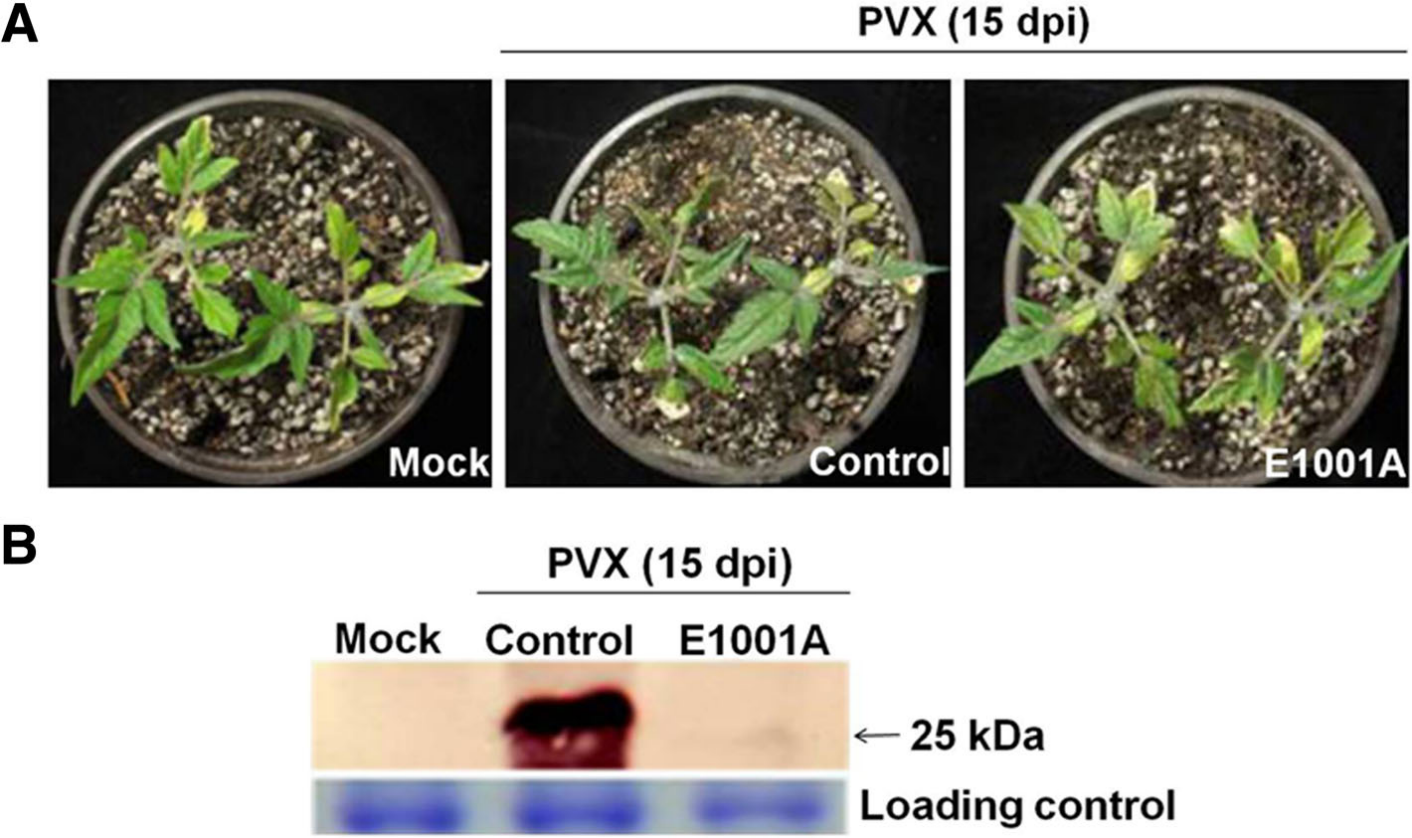
Cross protection of tomato plants with the E1001A against the challenge inoculation with PVX. **a** Tomato cv Micro-Tom plants pre- inoculated with empty vector pCAMBIA0390 (control) and E1001A, respectively, were challenged with PVX at 10 dpai. The symptoms were photographed at 15 days after the challenging inoculation. **b** Virus accumulation in the systemically infected tomato leaves were determined by Western blotting using antiserum against PVX CP at 15 days after the challenging inoculation. Total proteins were used as loading control

The above results showed that mutant E1001A provided better cross protection than any other attenuated mutants in *N. benthamiana* and *N. tabacum* plants. To examine whether E1001A was able to confer the cross protection against severe infection in other host plants, tomato plants cv. Micro-Tom were first inoculated with E1001A, and then challenge inoculated with wild type PVX at 10 dpai. No symptom was observed and only low concentration of PVX CP were detected by Western blotting at 15 days after challenging inoculation (Fig. 3a and b). Even at 25 days after challenging inoculation, all the plants pre-inoculated with E1001A still showed no symptoms (data not shown). The results showed that E1001A could provide complete protection against severe infection in tomato plants.

### Protection effect of a mutant with double mutations to severe infection

**Fig. 4:**
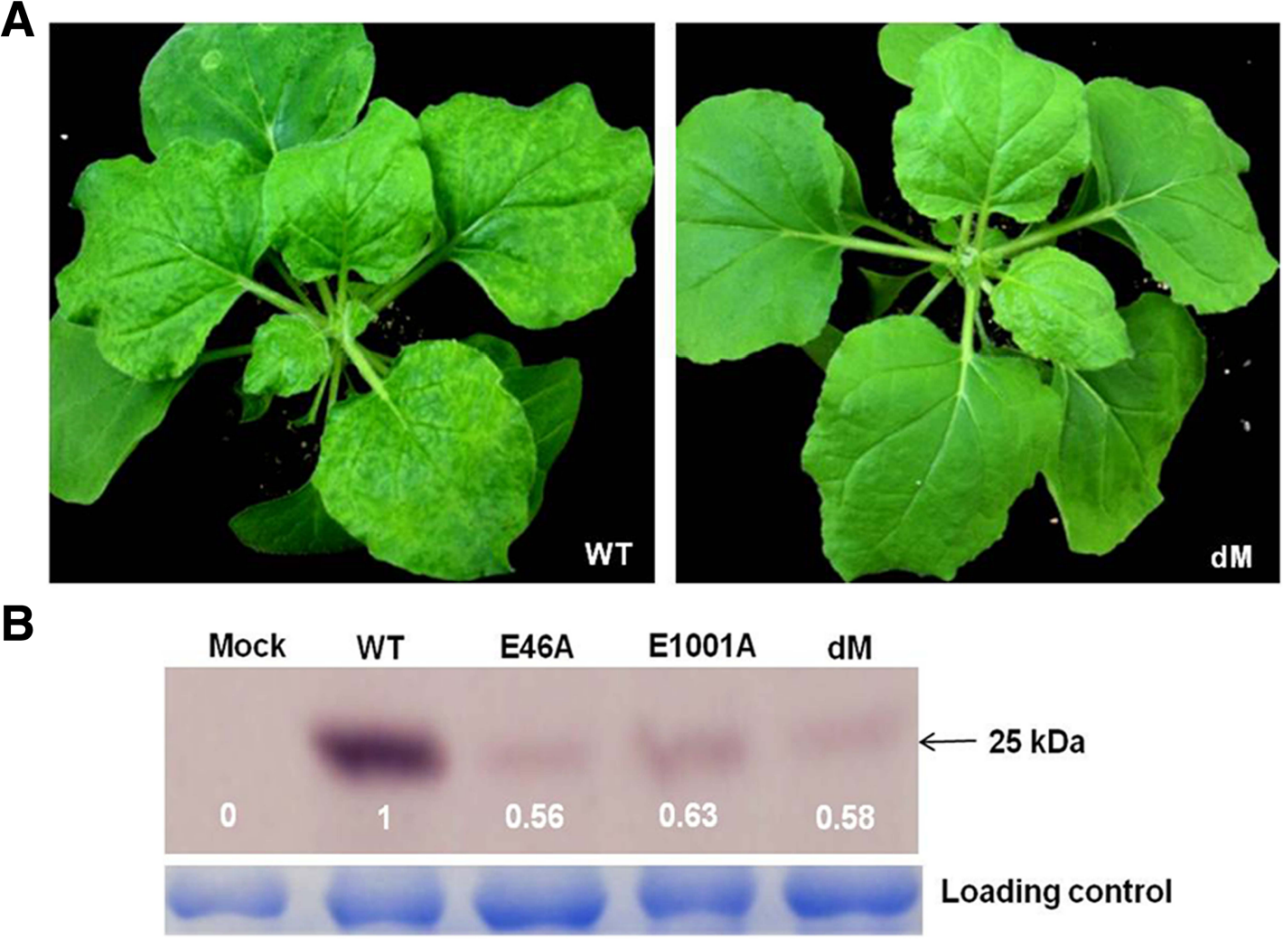
Symptom (**a**) and virus accumulation (**b**) of mutant dM with double substitutions in *N. benthamiana* plants at 20 dpai. Total proteins were used as loading control

To reduce the risk of reverse mutation, we constructed a new PVX mutant designated as dM (i.e. double mutations) in which both Glu^46^ and Glu^1001^ of RdRp were mutated to Ala. The mutant dM was still asymptomatic in *N. benthamiana* plants at 20 dpai (Fig. 4a), and accumulated CP to a level higher than that of E46A but lower than that of E1001A (Fig. 4b). Then we tested its ability to mediate protection against challenging with the wild type PVX. When the protective interval was ten days, all *N. benthamiana* plants pre-inoculated with dM showed severe symptoms on upper leaves and accumulated high level of CP at 25 days after challenging inoculation (Fig. 2a and b). But when the protective interval was increased to 15 days, of the 18 plants pre-inoculated with dM, only 13 *N. benthamiana* and 14 *N. tabacum* plants were systemically infected with the challenging virus (Table 2). These results indicated that mutant dM could provide incomplete protection against severe strain with a protective interval of 15 days.

### Genetic stability of attenuated mutants

The stability test results showed that, after successive transfer of 5 generations, neither of the *N. benthamiana* plants inoculated with mutants E1001A and dM showed obvious PVX symptoms. The sequencing results indicated that neither mutant E1001A produced recovery mutation after five successive passages in *N. benthamiana* plants, suggesting that the mutations at Glu^46^ and E^1001^ were stable genetically.

### Relationship between siRNA accumulation levels of attenuated mutants and efficacy of cross protection

To explore the relationship between siRNA accumulation levels of attenuated mutants and efficacy of cross protection, we used the attenuated mutants in the protective inoculation and pCaPVX440GFP in the challenging inoculation, and then detect the siRNA with different probes.

**Fig. 5:**
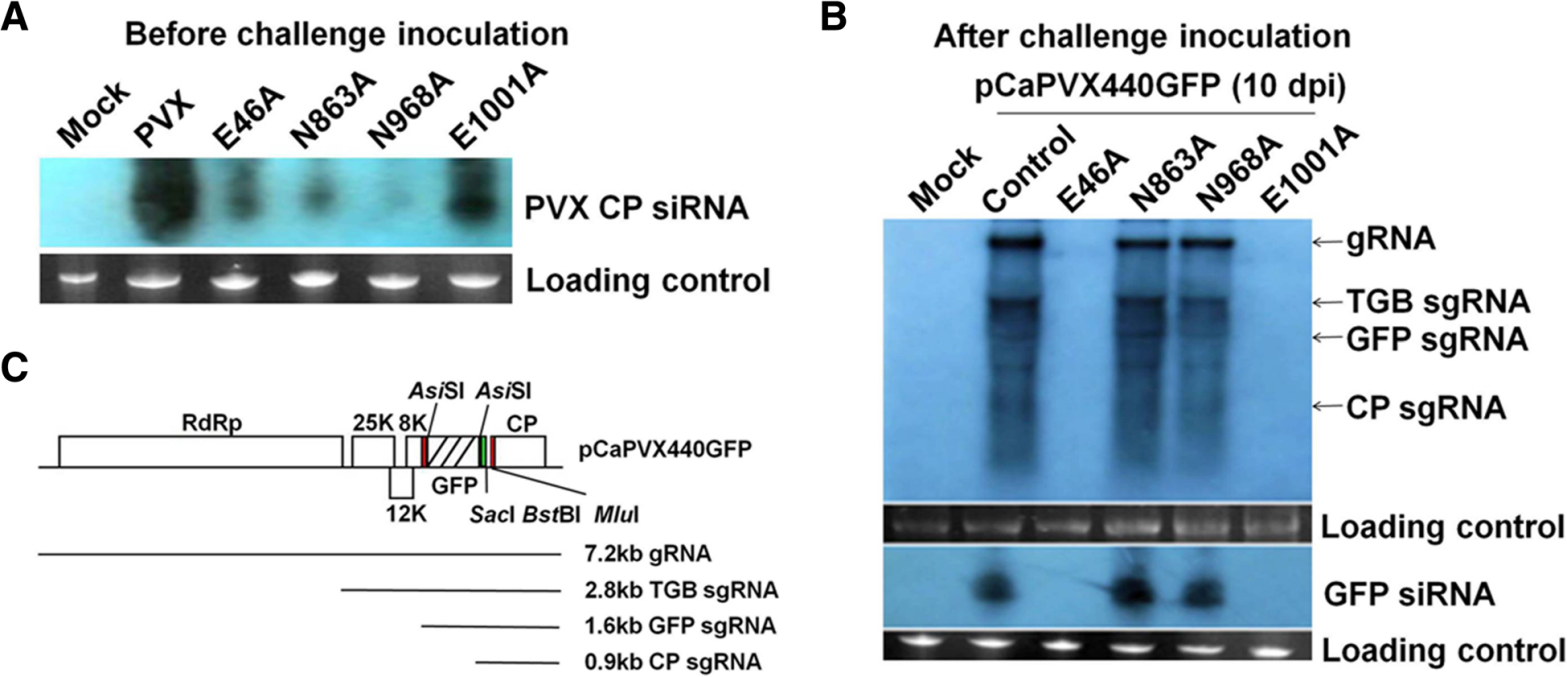
Accumulation of viral RNA and siRNA in *N. benthamiana* plants before and after challenging inoculation. **a** Accumulation of siRNA derived from WT PVX, mutants E46A, N863A, N968A, E1001A at 10 dpai. **b** Accumulation of virus-derived RNA and siRNA after challenge inoculation with pCaPVX440GFP in the same plants. The protective interval was 10 days. The RNA and siRNA were detected by Northern blotting hybridization at 10 days after challenge with saps from pCaPVX440GFP infected plants. **c** Genomic organization and approximate sizes of genomic and subgenomic RNAs derived from pCaPVX440GFP. The red rectangles indicated the PVX CP subgenomic promoter (SGP), green rectangle indicated the position of TMV CP SGP. The experiments were repeated three times independently. Total siRNAs were used as loading control

The accumulation levels of siRNA from different attenuated mutants were determined via Northern blotting assay using probe specific for the PVX CP gene. E1001A, which provided complete protection to the challenging virus, accumulated the highest level of siRNA, while mutant E46A which conferred incomplete protection against severe infection accumulated low levels of siRNAs. The mutants N863A and N968A which did not show any protection accumulated lower levels of siRNAs (Fig. 5a). These results indicated there existed a positive relationship between the siRNA accumulation levels and the levels of resistance mediated by the attenuated mutants.

When the protective interval was 10 days, high levels of viral RNAs accumulated in plants pre-inoculated with control plasmid, N863A and N968A, respectively, at ten days after challenging inoculation; however, no viral RNA was detected in plants pre-inoculated with E46A and E1001A (Fig. 5b). The 21–22 nt siRNAs specific for *gfp* gene were detected in *N. benthamiana* plants pre-inoculated with control, N863A and N968A, but not in plants pre-inoculated with E46A or E1001A (Fig. 5b), indicating that the invading virus derived from pCaPVX440GFP could not accumulate to detectable levels.

## Discussion

In this study we showed that the mutation of Glu^46^, Asn^863^, Asn^968^ or Glu^1001^ in RdRp to Ala reduced the virulence of PVX and that mutant E1001A displayed the highest cross protection efficacy against severe PVX infection in both *N. benthamiana* and tomato plants.

The aa residue at position 1422 of the RdRp was reported to be responsible for the symptom development of PVX in *Nicotiana* plants [53]. In this investigation, for the first time, we demonstrated that four aa, i.e. Glu^46^, Asn^863^, Asn^968^ and Glu^1001^ of RdRp are novel genetic determinants regulating the PVX virulence, and substitutions with alanine at any of these four residues significantly reduced the symptom severity of PVX (Fig. 1a). Mutations of negatively-charged residues Glu at position Glu^46^ and Glu^1001^ to non-polar Ala attenuated the symptoms of PVX. The mutation may change the polarity and/or conformation of RdRp, thus affecting its interaction with potential host factor(s) or other viral products. In other hand, the net charge of RdRp may be critical to maintain its normal function. Similar results were observed in the CP of ZYMV [54]. Furthermore, the substitution of basic Arg^180^ in helper component-proteinase with hydrophobic aa Ile resulted in mild symptoms in squash [24]. Both amino acids Asn^863^ and Asn^968^ of PVX RdRp are postulated glycosylation sites. The substitution of either Asn^863^ or Asn^968^ with Ala alleviated the symptom of inoculated plants, implying glycosylation at these two sites in RdRp is critical for PVX virulence. However, more work should be conducted to show if glycosylation occurs in vivo*.*

The symptom severity of some viruses was positively correlated with viral accumulation level in the infected leaves [45, 46], while that of other viruses was not [42, 44, 55]. The four PVX mutants induced similar mild symptoms but accumulated to different concentrations in the systemic leaves of inoculated host plant (Fig. 1a and b). The differences in virus concentration were positively related to their accumulation levels of viral genomic RNA (Fig. 1b and c). Further analysis indicated that the virus concentrations were positively related to the accumulation levels of plus- and minus- strand RNAs of PVX (Fig. 1b, c and d). It is hypothesized that mutations at these four aa reduced the binding of RdRp with minus strand RNA, thus reducing the accumulation of plus strand RNA, by which they influence the concentration of viral particles.

Cross protection can be separated into three stages: initiation, resistance, and maintenance [24]. It requires an interval between the inoculations of the protector and challenge virus, to allow the full establishment of protector virus in the plant [24, 56]. When the interval was 5 days, none of the four mutants could induce protection to PVX, because the systemic infection has not fully established. When the protective interval was increased to 10 days, E1001A reached a threshold; i.e., a level of extensive viral spread and accumulation sufficient to protect plant from virulent PVX infection. When the interval was 15 days, E1001A provided complete protection, while E46A provided incomplete protection to PVX. These results suggested that longer protective intervals could increase the cross protection efficacy of attenuated mutants against PVX.

The mechanisms underlying cross protection are not fully understood [56, 57]. The two major models for cross protection are CP mediation and RNA silencing mediation [56, 58]. The CP-mediated cross protection model is based on the finding that transgenic plants expressing TMV-CP or plants infected with PVX vector expressing TMV-CP are resistant to TMV infection, suggesting that the uncoating of the second strain was prevented [59, 60]. However, this hypothesis cannot explain the phenomena that CP-defective viruses and viroids can also confer cross protection. Then the RNA silencing model was suggested and accepted to explain the cross protection phenomenon for RNA and DNA viruses, as well as for viroids [56]. Plants pre-inoculated with E1001A and E46A displayed complete and incomplete resistance, respectively. The accumulation levels of siRNA were positively correlated with the efficiency of resistance (Fig. 5a and b). Plants inoculated with E46A had similar CP accumulation with that of N968A, however, E46A had much higher genomic and siRNA accumulations than that of N968A, which could explain why E46A had higher resistance efficiency than N968A (Fig. 1b and c; Fig. 5a and b). However, the mechanism underlying cross protection remains to be elucidated.

Based on results on a potyvirus *Turnip mosaic virus*, Kung et al. [57] proposed four criteria for the identification of a useful mild strain for effective cross-protection. Our results agree with their ideas in that the mild strain should produce stable mild symptoms and yet should not cause severe adverse effects on the protected host, and especially, a moderate and sustained titer of a protective virus is necessary to trigger cross-protection. The mutant E1001A accumulated highest level of CP and conferred highest cross protection efficacy. However, in this paper, we just introduced mutations to the RdRp of PVX, without changing the suppression activity of P25.

Among the methods available for mild strain screening, site-directed mutagenesis has unique advantages. Once an infectious clone of certain virus is constructed, it is easy to produce large number of mutants and evaluate their potential in cross protection. The data in this study and previous reports [16, 24] illustrated that the strategy we adopted here offers a rapid and effective way to create mild strains that have potential in cross protection. By modifying available mild strains of *Papaya ringspot virus*, the effectiveness of HA5–1 against heterologous isolates was improved [14]. By using mutagenesis, Kung et al. generated mild strain Tu-GK which provided complete cross-protection against *Turnip mosaic virus* in *N. benthamiana* and *Arabidopsis* plants [57].

The major risk of cross protection is that mild strains may become virulent ones by reverse mutations [3, 56]. One solution to reduce such risk is to create mild strains with two or more mutations. The mutant dM, which contains two mutations at both Glu^46^ and Glu^1001^, was asymptomatic and accumulated to a level lower than that of E1001A. It can provide protection against severe PVX infection in *N. benthamiana* and *N. tabacum* plants when the interval was 15 days. Genetic stability test showed that the both mutants E1001A and E 46A were stable genetically and did not show obvious symptom after successive transfer for five generations.

Cross protection is a promising strategy for biological control of plant viral diseases. The elucidation of viral virulence determinants via reverse genetics is an important step towards screening mild strains for cross protection. The findings of this research further confirmed the potential and validity of genetic engineering for producing mild strains and laid a solid foundation for the control of PVX via cross protection.

## Conclusions

The result in this study showed that substitutions of RdRp Glu^46^, Asn^863^, Asn^968^ or Glu^1001^ to Ala drastically attenuated PVX symptom and mutants E1001A and E46A could protect *Nicotiana benthamiana* and tomato from severe infection. Mutant dM which carries mutations in both Glu^46^ and Glu^1001^ provided effective resistance to wild type PVX. Both mutants E1001A and E46A had no recovery mutation after 5 successive transfers with interval of 10 days. However, further studies will be needed to test if the mutants will keep stable genetically after transferring for longer times, and to dissect the molecular mechanisms regulating cross protection. The findings of this research laid a solid foundation for the control of PVX via cross protection.

## Abbreviations

aa: Amino acids
CP: Coat protein
DIG: Digoxigenin
M-MLV: Moloney *Murine Leukemia Virus*
NC: Nitrocellulose
ORF: Open reading frame
PVX: *Potato virus X*
RdRp: RNA-dependent RNA polymerase
RT-PCR: Reverse transcription-polymerase chain reaction
SDS-PAGE: Sodium dodecyl sulfate-polyacrylamide gel electrophoresis
siRNAs: Small interfering RNAs
TBST: Tris-buffered saline containing 0.05% Tween-20
TGB: Triple gene block
TMV: *Tobacco mosaic virus*
